# Benefits from Vergence Rehabilitation: Evidence for Improvement of Reading Saccades and Fixations

**DOI:** 10.3389/fnint.2016.00033

**Published:** 2016-10-20

**Authors:** François Daniel, Aurélien Morize, Dominique Brémond-Gignac, Zoï Kapoula

**Affiliations:** ^1^IRIS Group, Physiopathologie de la Vision et Motricité Binoculaire Centre National de la Recherche Scientifique FR3636 Université Paris DescartesParis, France; ^2^Ophthalmology Service, Hôpital Necker - Enfants MaladesParis, France

**Keywords:** cognition, rehabilitation, reading saccades, vergence eye movements, fixation

## Abstract

We hypothesize that binocular coordination of saccades is based on continuous neuroplasticity involving interactions of saccades and vergence. To test this hypothesis we study reading saccades in young students who were diagnosed for vergence disorders before and after vergence rehabilitation. Following orthoptic evaluation and symptomatology screening, 5 weekly sessions of vergence rehabilitation were applied with the REMOBI vergence double step protocole (see Kapoula et al., 2016). Using the Eyeseecam videoculography device we measured vergence as well as saccades and fixations during a reading test four times: at the beginning and at the end of the first and of the fifth vergence rehabilitation session. The results show elimination of symptoms, improvement of clinical orthoptic scores, and importantly increase of measured vergence gain and reduction of inter-trial variability. Improvement of the vergence was associated to a decrease of the disconjugacy of saccades during reading but also to shortening of fixation durations, to reduction of the number of regressive saccades and to a better correction of the intra-saccadic disconjugacy during the following fixation. The results corroborate the hypothesis of neuroplasticity based on saccade vergence interaction in young adults. It validates the clinical validity of the vergence double-step REMOBI method as a means to improve both, vergence and reading performances. It opens a new research approach on the link between fine binocular coordination of saccades, quality of the vergence response, attention, cognition and reading.

## Introduction

The visual task of reading requires well-coordinated eye movements. To bring one word after another onto the fovea, the eyes have to execute several horizontal saccades while maintaining the angle of vergence stable, as reading is essentially a close distance activity, requiring precise fixation of the words. The task of reading needs quality of binocular coordination of the saccades, as poor binocular coordination of saccades during reading may result in fixation disparity (Evans, 2002; Blythe et al., 2006; Kliegl et al., 2006; Liversedge et al., 2006; Nuthmann and Kliegl, 2009; Jainta et al., 2010).

The quality of binocular coordination depends on vergence control and neuroplasticity. It is characterized by the measure of disconjugacy during and after the saccades, i.e., the difference of amplitude between the two eyes. Saccades in healthy adult humans are not perfectly well coordinated (see Kapoula et al., 1986; Collewijn et al., 1988). Indeed, because of the asymmetry of the oculomotor plant (difference in muscles and/or in innervations relays), a central saccade command concerning the two eyes (in obedience with Hering's law) could still result in a saccade that is slightly larger in the abducting than in the adducting eye (see Collewijn et al., 1988). Binocular coordination is monitored by learning and neuroplasticity, evidence for this was first presented by Fioravanti et al. (1995), who reported that binocular coordination of saccades is poor in young children, but improves with age. Interestingly, Yang and Kapoula (2003) also found that the disconjugacy during saccades in children was much more variable than that for adults. Similar results have been shown during a reading task (Blythe et al., 2006). Therefore, the development of binocular coordination appears to be driven by visual experience and is based on neural plasticity/maturation. Yang and Kapoula (2003) hypothesized that the central nervous system learns to couple together with the saccade a fast vergence command that helps to reduce the abduction adduction asymmetry, at near vision, when the eyes are already maintaining convergence angle, learning to produce appropriate intra-saccadic convergence appears to be more difficult.

In the context of this theoretical framework, we hypothesized that ocular motor training improving vergence and accommodation in persons with vergence and accommodation disorders should lead to a benefit for the binocular coordination of saccades. Orthoptics training not only increases vergence capacities and reduces the number of symptoms (Griffin, 1987; Von Noorden, 2002; Cooper and Feldman, 2009; Scheiman et al., 2009), but also improves binocular coordination during and after the saccades (Bucci et al., 2006). This was shown for children with vertigo and vergence disorders. Gaertner et al. (2013) also showed that traditional orthoptic training objectively improved vergence performances and the binocular coordination of saccades during reading comparing saccades before and after orthoptics, with decrease of their disconjugacy. However, the texts used were short (single sentence), and consequently the appraisal of the reading speed and of the quality of coordination over time could not be investigated with precision.

Subjects showing convergence insufficiency (CI) and undergoing vergence rehabilitation were the focus of the recent studies of Alvarez et al. (2010, 2014) and Alvarez and Kim (2013). The main results showed that before the vergence training, CI subjects showed less symmetrical peak velocity between the left and the right eye than controls. Another fMRI study by the same authors examined functional activity of the cortical areas such as Frontal Eye Field (FEF), Posterior Parietal Cortex (PPC), and Cerebellar Vermis (CV), which are implicated in convergence programming: activity was reduced in CI subjects compared to controls. Moreover, after the vergence training, the peak velocity was significantly more symmetrical than before in CI subjects and this was correlated to the increase in the functional activity within the FEF, the PPC and the CV. The authors propose that saccade may be a compensatory mechanism used by CI subjects when convergence peak velocity is reduced. This idea is also compatible with the concept of neural plasticity and vergence and saccades interactions.

The goal of the present study is to test to what extent rehabilitation of vergence eye movement along the medial plan would improve the quality of binocular coordination of saccades when reading in young adults diagnosed with vergence disorders. Orthoptic training is based on empirical exercises (such as pen push-ups, or prism bar exercises) and the practice can vary from one practitioner to the other. A major attribute of the present study is that vergence disorder is rehabilitated with a research based method (Double step vergence protocol with the REMOBI device, see **Figure 2**; Kapoula et al., 2016). A precise rehabilitation protocol is applied with targets in the real 3D space, designed with specific time and space constraints. Rehabilitation involved a total amount of 2200 vergence movements performed in 5 sessions of 35 min each.

Dusek et al. (2011) showed that reading speed increased significantly in children diagnosed with CI who were treated with orthoptics in contrast to children who did not follow orthoptics. Increase of reading speed could have been inducted by shorter fixation duration but this was not investigated in this study. A second objective of the present study is to check if and how rehabilitation of vergence could eventually improve not only the binocular coordination of saccades but also other parameters such as fixation duration that reflects cognitive process. Improving vergence capabilities could improve cognitive executive functions (Daniel and Kapoula, 2016).

A third objective is to focus on both, the short term and the long term effects of vergence rehabilitation and their consequences on reading, saccades and fixations. Short term rehabilitation effects have been reported in studies of binocular coordination of saccades (Eggert and Kapoula, 1995; Kapoula et al., 1995). For instance, it was demonstrated the existence of a short term adaptive saccade disconjugacy using a magnifier glass (10%) in front of one eye. In the present study, we investigate if a short term training of 35 min of vergence could influence the quality of binocular coordination of saccades and fixations immediately afterwards. We will call “short term effects” the effects occurring within a single session of rehabilitation, i.e., between the two reading trials run before and after the same session of vergence rehabilitation. We will also search for “long term effects” by comparing reading trials before and after the complete rehabilitation program, consisted of 5 weekly sessions of 35 min each.

## Materials and methods

### Subjects

Nine students (4 males, 5 females, aged 25.7 ± 9.7) diagnosed with vergence disorders by orthoptic examination (for more details, see Kapoula et al., 2016), showing high symptomatology scores (≥20 at the Convergence Insufficiency Symptom Survey, CISS, Rouse et al., 2004) and recruited mostly in a technical secondary school specialized in optics (*Lycée Fresnel*, Paris), underwent a rehabilitation of vergence eye movements. Training was done once a week at a rate of 35 min each time, during 5 weeks (for more details, see Kapoula et al., 2016). We also tested 3 additional control subjects (2 males, 1 female, aged 21 ± 1) for one session of reading tests.

The investigation adhered to the tenets of the Declaration of Helsinki and was approved by the local human experimentation committee, the “Comité de Protection des Personnes” (CPP) Ile de France VI (No: 07035), Necker Hospital in Paris, France. Informed written consent was obtained from subject.

### Orthoptic screening

Orthoptic screening was done for all the subjects before the rehabilitation started, on separate days. A second orthoptic examination was done 1 month after the end of the vergence training, on a different day, to evaluate the evolution after the treatment in different areas. Subject 4 missed the last examination. Major results are shown on Table 1.

**Table 1:** **Orthoptic screening**.

	**NPC**			**Divergence range**		**Convergence range**		**CISS score**	
**Subject**	**Before**	**After**		**Before**	**After**	**Before**	**After**	**Before**	**After**
1	5	5	Far	4	4	10	16	21	19
			Near	10	10	30	30		
2	8	5	Far	16	16	18	25	30	13
			Near	18	18	35	35		
3	6	5	Far	8	8	10	40	35	4
			Near	12	12	16	40		
4	15	–	Far	6	–	6	–	34	17
			Near	10	–	12	–		
5	5	3	Far	4	4	14	25	32	21
			Near	6	6	30	40		
6	5	6	Far	4	6	16	16	13	9
			Near	8	8	20	25		
7	5	5	Far	6	4	14	40	32	31
			Near	14	12	25	25		
8	5	5	Far	6	10	20	20	26	12
			Near	12	14	25	25		
9	10	5	Far	6	6	8	20	25	12
			Near	6	14	12	18		
Mean (±SD)	7 (±3)	5 (±1)	Far	7 (±4)	7 (±4)	13 (±5)	25 (±10)	27.6 (±7.1)	15.3 (±7.8)
			Near	11 (±4)	12 (±4)	23 (±8)	30 (±8)		

### Reading test, vergence tests, and eye movements recording

**Figure 1:**
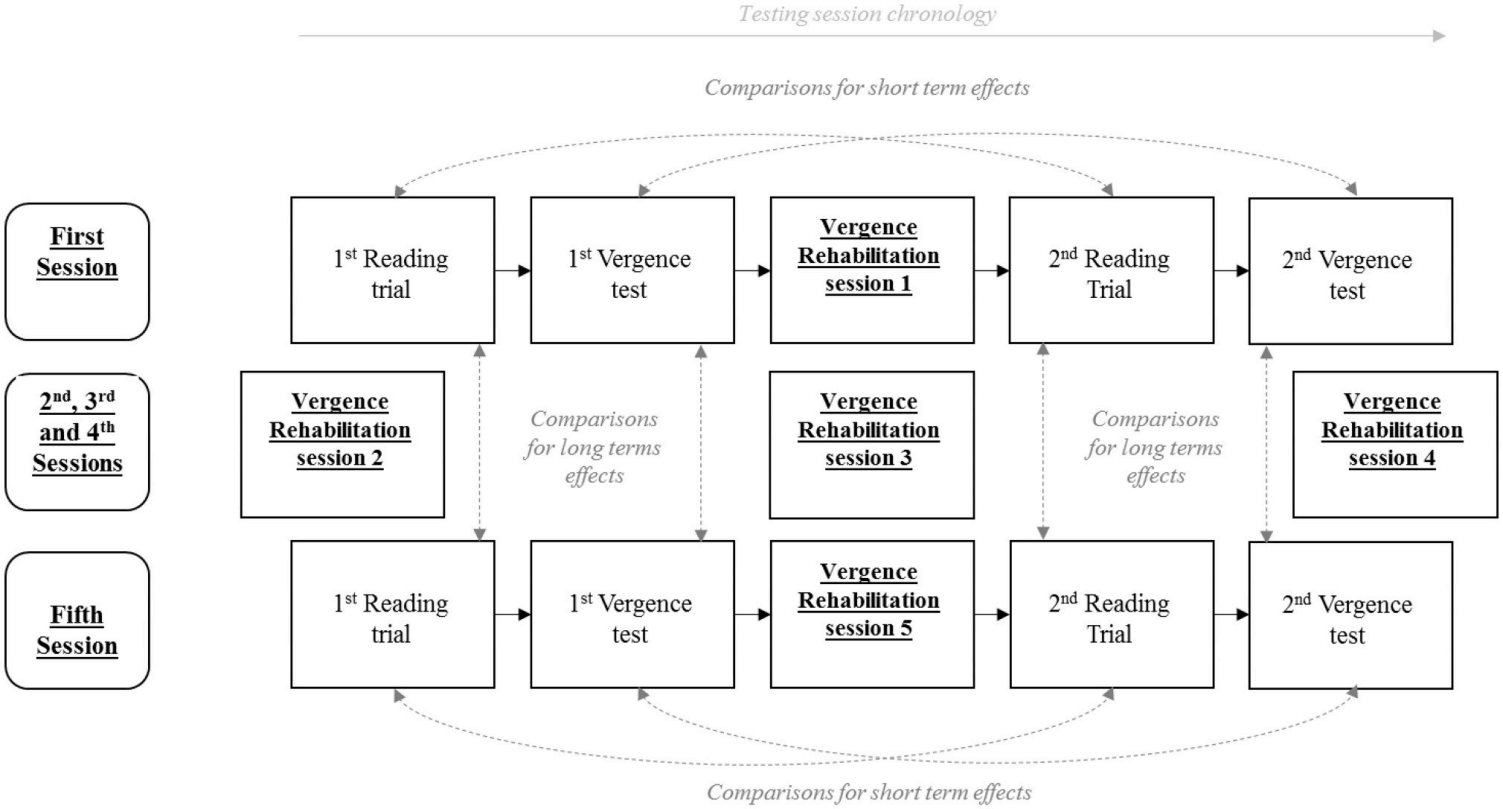
**Testing chronology: positioning of each test during the first and the fifth vergence rehabilitation session**.

Four trials of a reading test were recorded: the first and the second ones respectively at the beginning and the end of the first session of rehabilitation run with the REMOBI device, and the third and the fourth ones respectively at the beginning and the end of the fifth session of rehabilitation. The organization of all the sequences of reading tests, vergence tests and vergence training sessions is shown on Figure 1.

#### Reading test

**Figure 2:**
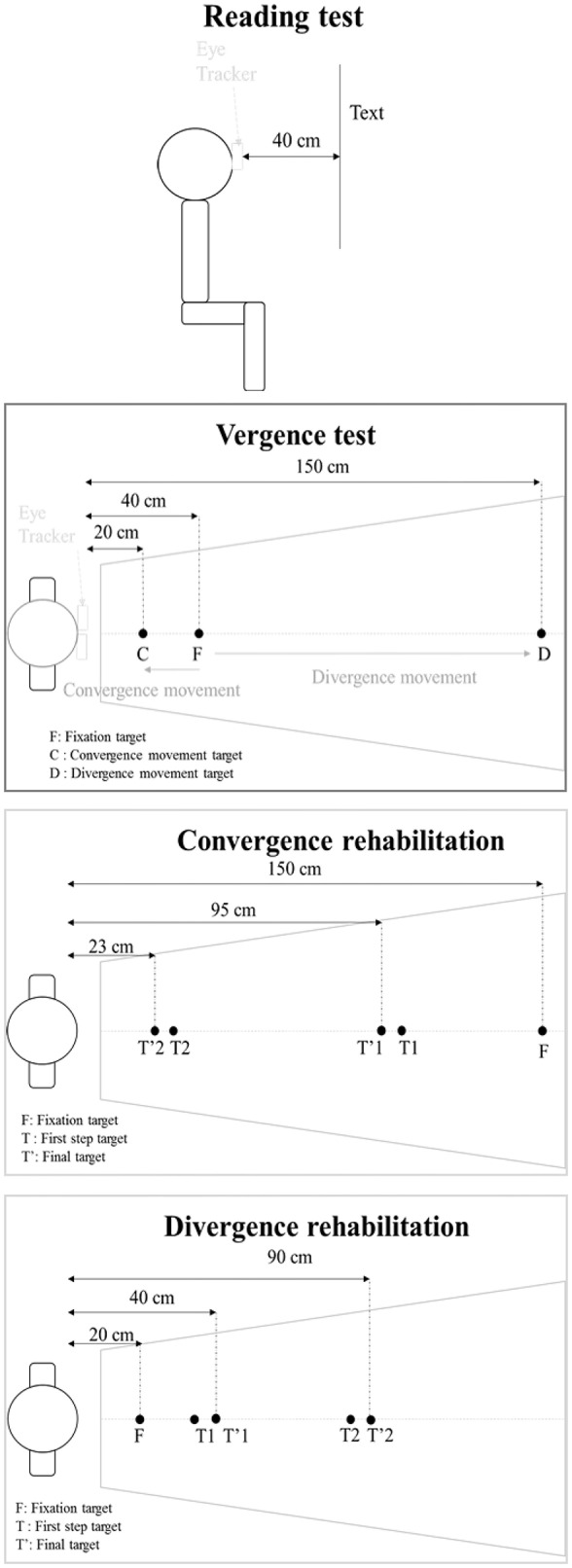
**Testing protocol: the reading test and the vergence test using the REMOBI, accomplished before and after the first and the fifth session of vergence rehabilitation, and the convergence and divergence rehabilitation using the REMOBI and the double step vergence protocol, accomplished at every session of rehabilitation**.

We asked every subject to read silently a whole text at their own speed and raise a finger when they had finished. We used the Alouette test, a 265-word text in French (Lefavrais, 1967; Debray et al., 1972), firstly developed for children suspected of dyslexia, to evaluate their basic reading skills, but not their comprehension or memory. It was printed in courier font size 12 and each letter was about 0.3° of angular size, and was fixed on a wall, in front of the sited subject, at 40 cm distance (see Figure 2). The reading test was chosen considering the repeatability, as we wanted to limit the training effect. Even if the Alouette test was basically designed to diagnosed problems of reading like dyslexia in children and therefore implicates to read it aloud, this text is interesting as it is composed with sentences that are not connected with each other and with non-common words. As a result, this text doesn't have any meaning, and memorization or anticipation are not facilitated. It permits an evaluation of the automatic task of reading. Objective eye movement recording is therefore important to ensure that every word of the Alouette text was fixated and the analysis of the saccades parameters, in terms of numbers or amplitude, will give us more information on the behavior of the subjects during a silent reading task.

#### Vergence test

The REMOBI device was run with the vergence test. A fixation LED appeared first to a location of 40 cm during 2000 ms, and was followed by a second target, appeared 1200–1800 ms after the fixation one and randomly located further or closer from the fixation LED, respectively at 150 or 20 cm, during 2000 ms. Each vergence test contained 40 trials, 20 for convergence movements and 20 for divergence movements (see Figure 2).

#### Vergence rehabilitation protocol

Subjects performed 5 weekly sessions of 35 min vergence rehabilitation using the Vergence double-step protocol with a patented REMOBI device (US 8851669); the REMOBI is composed by a trapezoid surface of LEDs and adjacent buzzers.

A double step-paradigm was used in the convergence and the divergence rehabilitation trials: a fixation target appeared first to a location (150 cm for convergence trials, 20 cm for divergence trials) during 1600 ms, and was immediately followed by a second target (randomly located at 99 or 26 cm for convergence trials and 34 or 57 cm for divergence trials) during 200 ms and a final target (respectively located at 95 or 23 cm for the convergence trials and 40 or 90 cm for the divergence trials) during 1300 ms (see Figure 2, for further details see Kapoula et al., 2016). The vergence double-step protocol was designed to expose the visuo-motor system to an error that cannot be corrected online and leads to adaptive readjustment of the gain of the motor control. Takagi et al. (2001) were the first to show its validity for vergence as well in healthy subjects.

#### Eye movements recording

Eye movements were recorded during the vergence tests and during the reading trials, four times: at the beginning and the end of the first and the fifth session (see Figure 1). These data are different from those of the previous study of Kapoula et al. (2016) which presented results before a week and a month after vergence rehabilitation; the present study focuses on the recordings of vergence and reading during the first and the fifth vergence training session. The subject was asked whether to read the text or to fixate the LEDs, and eye movements were recorded binocularly with a video-oculography EyeSeeCam system (University of Munich Hospital, Clinical Neuroscience, Munich, Germany, see http://eyeseecam.com/). The sampling rate of the EyeSeeCam system was 222 Hz and the optimal spatial resolution was approximately 0.01°. At the beginning of the reading task, a 5 points calibration sequence was run using a matrices of laser dots: a central dot and four peripheral dots displayed at 8.5° rightward, leftward, downward and upward. Subjects fixated each dot one by one for four times, and total calibration task lasted less than 30 s. At the beginning of the vergence tests, a further calibration task was performed using the REMOBI to elicit 16 interleaved leftward and rightward saccades to LEDs located at 10° and 20° from the midsagittal plan, at a distance of 1.5 m from subjects' eyes.

### Eye movement analysis

Calibration factors for each eye were extracted from the saccades recorded in the calibration task. From the individual calibrated eye position signal we derived the horizontal conjugate signal by calculating the mean of the two horizontal eye positions, i.e., (left eye + right eye)/2, and the horizontal disconjugate signal by calculating the difference position between both eyes, i.e., left eye–right eye. The velocity of the horizontal conjugate and disconjugate signals were computed using a symmetrical two-point differentiator combined to low-pass filtering with a Gaussian Finite Impulse Response (FIR) filter (cut-off frequency 33 Hz).

**Figure 3:**
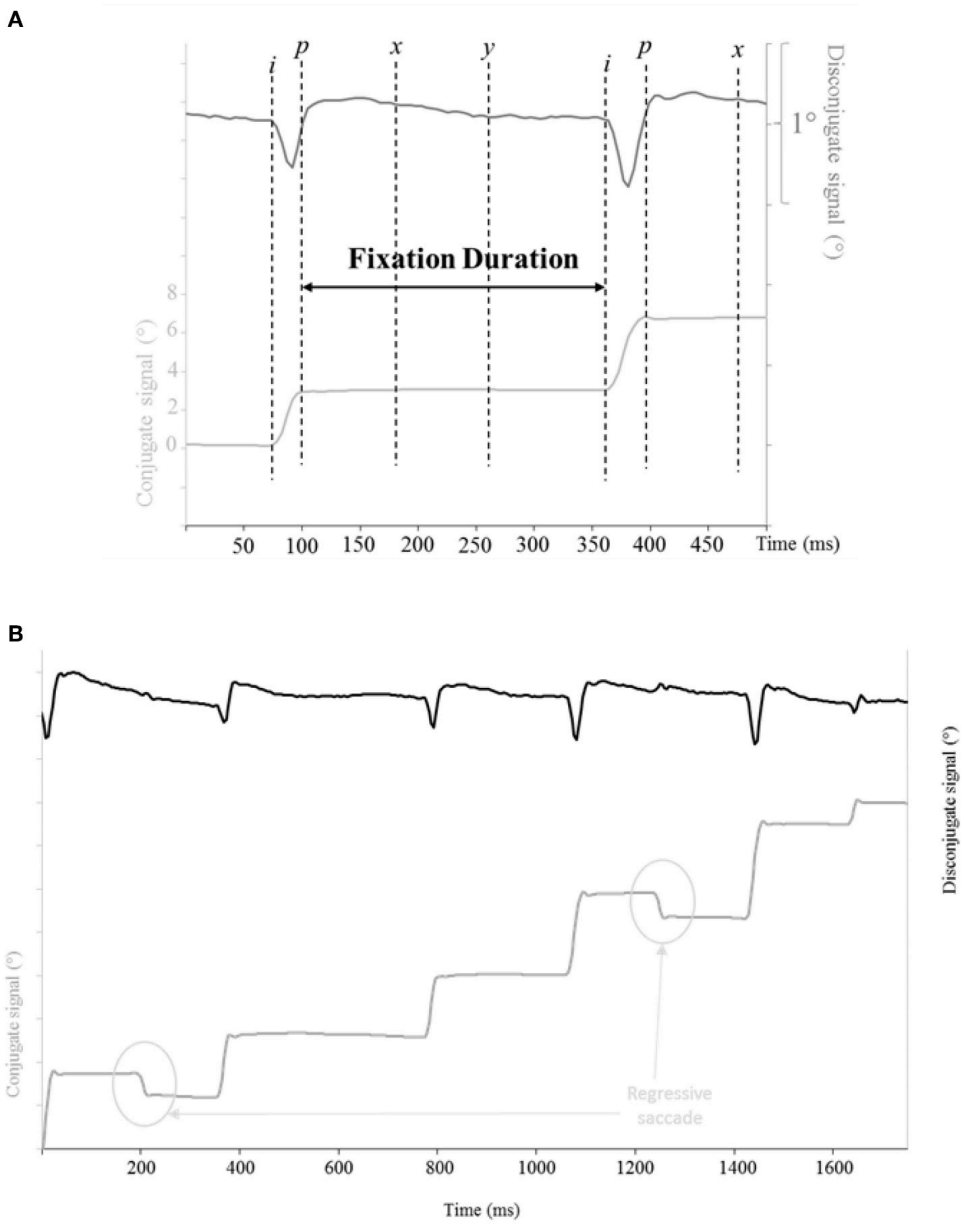
**(A)** Analysis and marking of the reading saccades: determination of the saccade and of the fixation duration. *i* and *p* indicate respectively the beginning and the end of each saccade. We studied the post-saccadic drift 80 and 160 ms after the end of the saccade, *x* and *y* indicate respectively these two periods of fixation. Lower trace: horizontal conjugate position. Upper trace: horizontal disconjugate position. **(B)** Evolution of the conjugate and the disconjugate position of the eyes over time when reading an entire line (subject 2). Lower trace: horizontal conjugate position. Upper trace: horizontal disconjugate position.

Horizontal eye movements were defined using the velocity of the signal, respectively conjugate velocity for saccades and disconjugate velocity for vergence. The onset, or offset, were marked as the time when velocity signal exceeded, or dropped respectively below 10% of the maximum velocity. Similar criteria have been used in several other studies (Bucci et al., 2001; Yang and Kapoula, 2003; Vernet and Kapoula, 2009): *i* for the onset and *p* for the offset of each eye movement (see Figures 3A,B). The automatic position of the markers was carefully verified by visual inspection of the individual eye movement traces. From these markers, we measured the amplitude of the movement (between *p* and *i*). Some eye movements were rejected from the analysis.

### Analysis of the reading test

A few markers were added from the saccades analysis, *x* and *y* respectively 80 and 160 ms after *p*, as post-saccadic fixation marks (see Figure 3A).

From these markers, we measured the amplitude of the post-saccadic drift during the first 80 ms (between *x* and *p*) and the first 160 ms (between *y* and *p*) of fixation, using the conjugate signal. We then calculated the disconjugacy (change of disparity) during the saccades (between *p* and *i*), during the first 80 ms of the post-saccadic drift (between *x* and *p*) and during the first 160 ms of the post-saccadic drift (between *y* and *p*), using the disconjugate signal. We also calculated the fixation duration (between *p* and the *i* for the next saccade, see Figure 3A).

For eight subjects, 80–90% of trials were used for statistical analysis, 10–20% were rejected due to blinks or partial lost signal during the recording; for one subject in one reading trial, 40% of the saccades were rejected due to a loss of signal for one eye during the recording.

### Analysis of the vergence test

**Figure 4:**
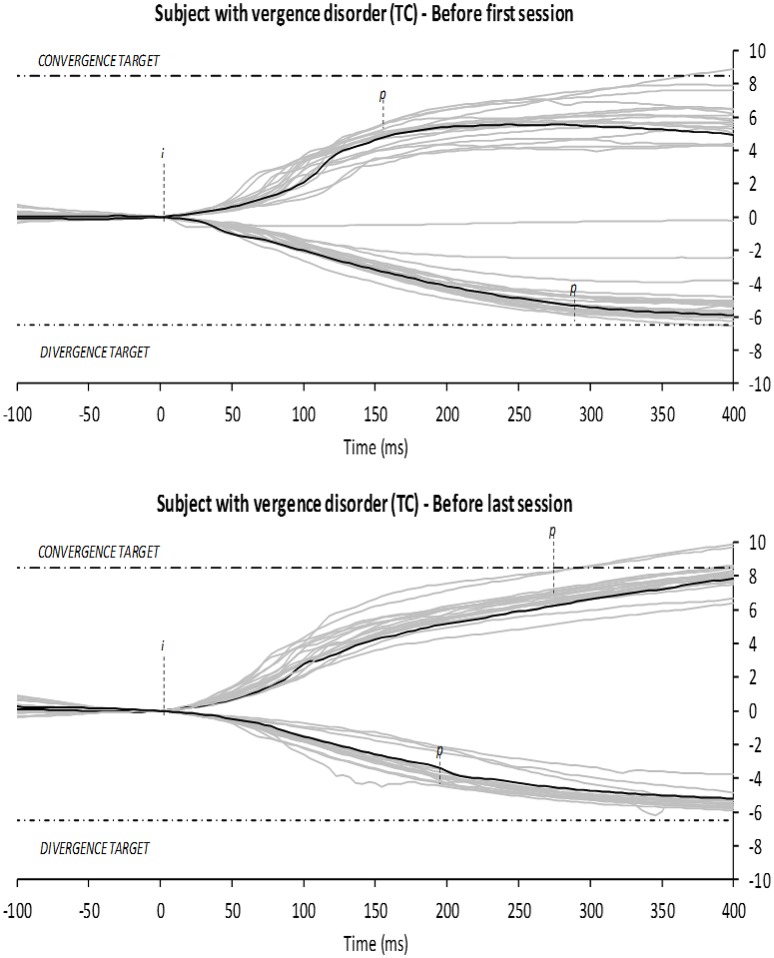
**Vergence test: disconjugate signal (subject 2) during the vergence test before the first session of rehabilitation and before the fifth session of rehabilitation**. *i* and *p* indicate respectively the onset and the offset of the vergence movement of the sample colored in black, the time point when the eye velocity exceeded or dropped below 5°/s. Superposition of each convergence or divergence trial is shown in gray.

The main parameter of the vergence analysis is the amplitude of the movement which was expressed as the gain, i.e., objective amplitude of the vergence response/requirement and its variability (see Figure 4). Pupillary distance was used to calculate the exact vergence requirement for each subject individually. It is important to note that most of the subjects diagnosed with vergence disorders showed strong abnormalities in convergence and/or divergence movements in terms of gain compared to control subjects (see Kapoula et al., 2016). For all subjects 75–100% of convergence and divergence trials were used in the statistical analysis. To be precise, 11.6 ± 8% were rejected mostly due to blink and loss of signal, similarly for convergence and divergence, at the group level which is similar to the rejection level from a previous study of overlap vergence in healthy young adults (Lang et al., 2014).

### Statistical analysis

We used the statistic program *Statistica* to perform our analysis. A non-parametric Friedman ANOVA was used as the number of subjects was limited; this analysis was applied on individual measures: as four different trials per subject have been run, we were looking for an effect of the vergence rehabilitation on the global time of reading, on the binocular coordination of the reading saccades and fixations, and on the temporal parameters of the fixations.

When a significant main effect of the vergence rehabilitation was found, a non-parametric Wilcoxon test was then performed to compare reading trials two by two: (1) to establish the short term effects of vergence training, we compare the first with the last reading trial or vergence test run within the same session, and this for each of the two session, before and after vergence training; (2) to establish the long term effects of rehabilitation, we compared the first reading trial of the vergence test of the session prior to rehabilitation with the first reading trial of vergence test of the session after the complete rehabilitation program was achieved, similarly we compared the last reading trial of the session prior to rehabilitation with the last reading trial of the session after the achievement of rehabilitation (see Figure 1). To double check the validity of the Friedman ANOVA results, we also performed a two-way ANOVA after data ranking, considering together the short term and the long term effects.

As the saccade disconjugacy can be corrected by the post-saccadic drift disconjugacy (see Vernet and Kapoula, 2009), we also searched for a possible correlation between the amplitude of the saccade disconjugacy and the amplitude of the post-saccadic drift disconjugacy. We used a Spearman correlation analysis on individual data and on the entire data set for each reading test.

As the global time of reading could be influenced by several temporal and motor parameters, we performed a multiple regression analysis to know which parameter between fixation duration and saccade disconjugacy could predict the best the global reading improvement.

## Results

### Effects of vergence rehabilitation on clinical tests

Vergence rehabilitation led to significant improvements of both orthoptic findings and CISS scores for the symptomatology (see Table 1). Statistically significant differences were found for the convergence at far distance and CISS Scores, the means being 13 ± 5 Prism Diopter (PD) vs. 25 ± 10 PD (*Z* = 2.37, *p* < 0.02) and 27.6 ± 7.1 vs. 15.3 ± 7.8 (*Z* = 2.67, *p* < 0.01) before and after vergence rehabilitation respectively. The mean value of the Near Point of Convergence (NPC) decreased and the mean value of the near convergence at near increased after the vergence rehabilitation, but these positive changes did not reach statistical significance (*p* > 0.05).

### Vergence tests

Mean values and standard deviations for the group of nine subjects are shown on Table 2, for each of the following parameters: gain and variability for the convergence and divergence movements.

**Table 2:** **Mean values and SD for the gain and the variability in the vergence tests**.

	**First test first session**	**Last test first session**	**First test fifth session**	**Last test fifth session**
**CONVERGENCE**				
Mean gain value	0.68^*^ (±0.2)	0.66^*^ (±0.2)	0.92^*^ (±0.2)	0.94^*^ (±0.17)
Mean variability value	37 (±23)	29^*^ (±22)	14 (±6)	13^*^ (±6)
**DIVERGENCE**				
Mean gain value	0.57 (±0.22)	0.69^*^ (±0.13)	0.75^+^ (±0.11)	0.81^+^^*^ (±0.14)
Mean variability value	36^*^ (±30)	17^*^ (±10)	14^*^ (±7)	13^*^ (±11)

* *Significant long term effect (between beginning of the 2 sessions or between end of the 2 sessions)*.

+ *Significant short term effect (between the first and the second attempt of one session)*.

#### Short terms effects

For the first session, no significant difference has been found (*p* > 0.05), neither for convergence or divergent movements. Yet, a significant difference has been found (*Z* = 2.43, *p* = 0.015) in terms of gain in divergence movements between the two tests for the final session (0.75 ± 0.11 vs. 0.81 ± 0.14), run after the complete program of vergence rehabilitation.

#### Long terms effects

##### Convergence movements

Significant increase of the gain has been found, between the first vergence test run before rehabilitation and the first vergence test run in the final session after the rehabilitation (0.68 ± 0.20 vs. 0.92 ± 0.20, *Z* = 2.31, *p* = 0.021); also between the last vergence test run after the first session of rehabilitation and the last vergence test run after the fifth session of vergence rehabilitation (0.66 ± 0.20 vs. 0.94 ± 0.17, *Z* = 2.67, *p* = 0.008). Significantly lower variability in the movements' amplitude has been found when comparing the last vergence test run after the first session of vergence rehabilitation with the last vergence test run after the final, fifth session of vergence rehabilitation (29 ± 22 vs. 13 ± 6, *Z* = 2.07, *p* = 0.038).

##### Divergence movements

Significant increase of the gain has been found when comparing the last vergence test run after the first session of vergence the rehabilitation and the last vergence test run before the final, fifth session of rehabilitation (0.69 ± 0.13 vs. 0.81 ± 0.14, *Z* = 2.19, *p* = 0.028). Significantly lower variability in the movements' amplitude has been found when comparing the vergence test run before the first session of vergence rehabilitation and the first vergence test run during the final, fifth session of vergence rehabilitation (36 ± 30 vs. 14 ± 7, *Z* = 2.07, *p* = 0.038); similarly when comparing the last vergence test run in the first and in the fifth session of vergence rehabilitation (17 ± 10 vs. 13 ± 11, *Z* = 2.31, *p* = 0.021).

**Figure 5:**
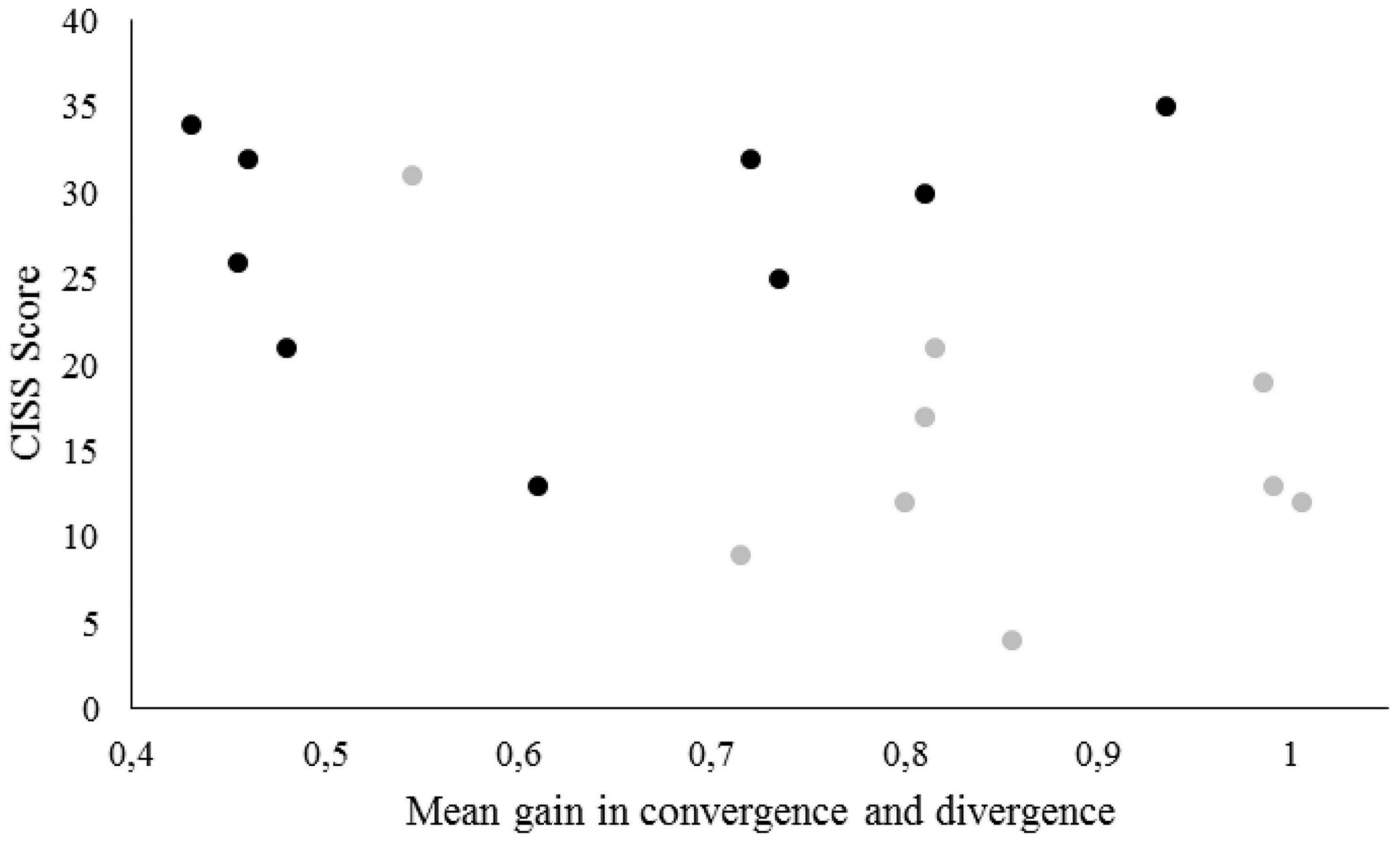
**Scatter plot of the mean gain values ((summation of the gain in convergence and divergence)/2) in the first vergence test of the first and the fifth session of rehabilitation as a function of the CISS score**. Black plots represent the gain values in the first vergence test of the first session as a function of the CISS score prior to rehabilitation. Gray plots represent the gain values in the first vergence test of the fifth session as a function of the CISS score after the vergence rehabilitation.

We compared the evolution of both CISS score and gain in convergence and divergence before and after the vergence rehabilitation. The decrease of the CISS score after the vergence rehabilitation was not significantly correlated with the increase of the vergence gain (p > 0.05, using a Spearman correlation analysis; see Figure 5).

### Reading tests

#### Reading saccades parameters

**Figure 6:**
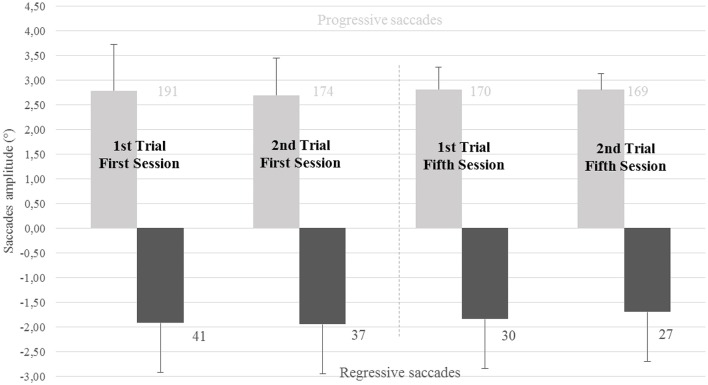
**Mean amplitude, SD and number of progressive and regressive saccades: in light-gray, the mean value and the standard deviation concerning the amplitude of the progressive reading saccades; in dark-gray, the mean value and the standard deviation concerning the amplitude of the regressive saccades**. Numbers on each column indicate the number of saccade in each reading trial next to each bar.

**Figure 7:**
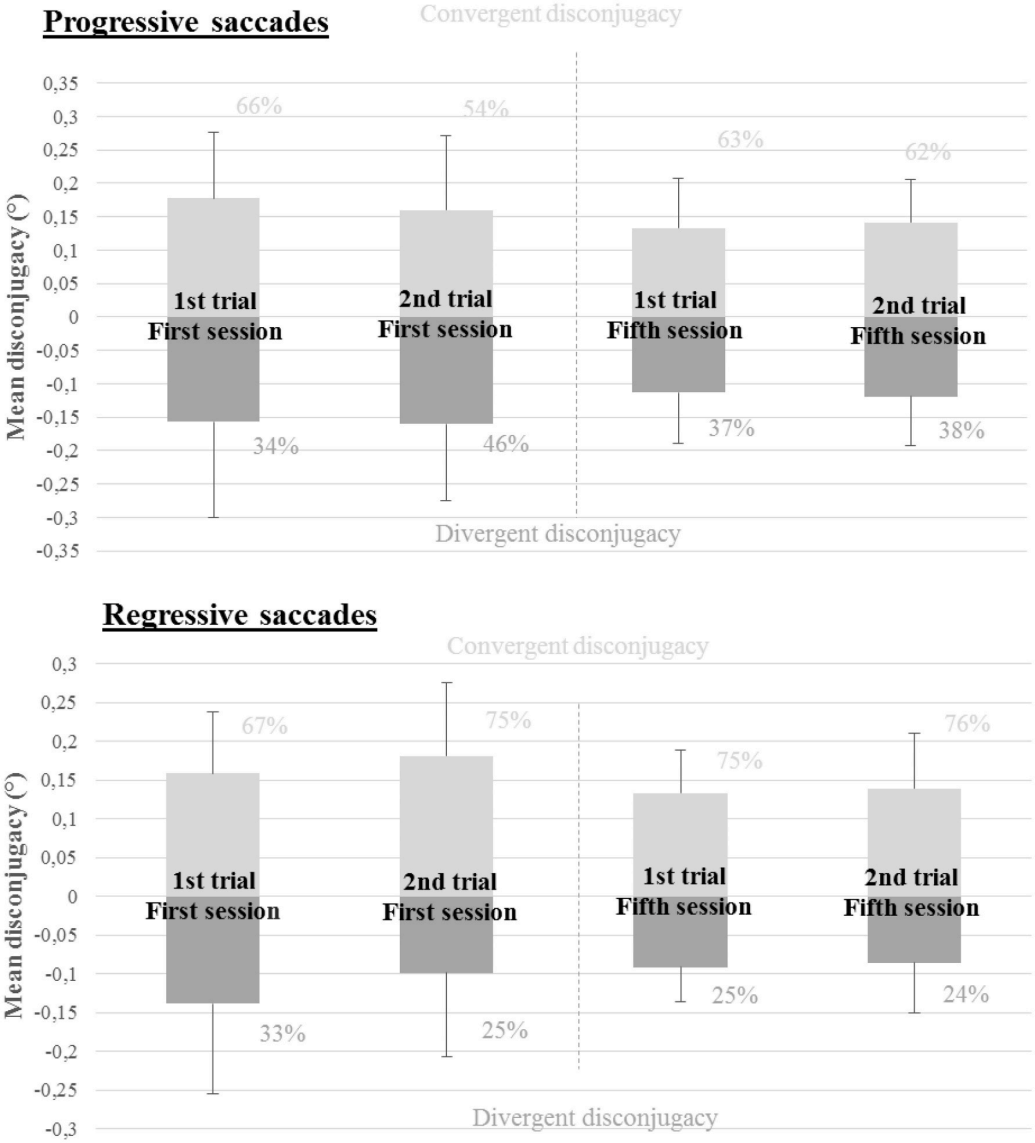
**Saccade disconjugacy in progressive and regressive reading saccades: in light-gray, the mean amplitude and standard deviation concerning the positive saccade disconjugacy; in dark-gray, the mean amplitude and standard deviation concerning the negative saccade disconjugacy**. Percentage of positive and negative saccade disconjugacy is indicated for each reading trial next to each bar.

Numbers, amplitude in degrees and standard deviation of progressive and regressive saccades are shown on Figure 6. Overall progressive saccades were of a higher amplitude than the regressive saccades (2.77 ± 0.05° vs. 1.85 ± 0.11°). Regressive saccades (see Figure 3B) were less frequent than progressive saccades and represented 16.11% of the total amount of saccades accomplished by the subjects. Mean values for the group of nine subjects in each reading trial concerning saccades amplitude, duration of the fixations, disconjugacy of the saccades and post-saccadic disconjugate and conjugate signals are shown on Table 3A for progressive saccades and on Table 3B for regressive saccades. Details about saccade disconjugacy results are shown on Figure 7 for progressive and regressive saccades.

**Table 3:** **Mean values and SD for the progressive and the regressive reading saccades parameters, and for the global reading time**.

	**First trial first session**	**Last trial first session**	**First trial fifth session**	**Last trial fifth session**
**(A)—PROGRESSIVE READING SACCADES**				
Saccades amplitude (°)	2.78 (± 0.93)	2.69 (± 0.75)	2.81 (± 0.45)	2.80 (± 0.33)
Fixation duration (ms)	286.46^*^ (± 59.97)	269.92^*^ (± 51.08)	266.79^*^ (± 56.70)	258.33^*^ (± 55.38)
Disconjugacy of saccades (°)	0.05 (± 0.17)	0.01 (± 0.15)	0.03 (± 0.13)	0.03 (± 0.13)
Disconjugacy of saccades (°) Absolute value	0.21^*^ (± 0.10)	0.19 (± 0.09)	0.15^*^ (± 0.08)	0.16 (± 0.07)
Disconjugate Post-saccadic drift 80 ms (°)	−0.02 (± 0.09)	−0.03 (± 0.07)	−0.02 (± 0.09)	−0.01 (± 0.08)
Disconjugate Post-saccadic drift 80 ms (°) Absolute value	0.12 (± 0.06)	0.11 (± 0.05)	0.09 (± 0.04)	0.10 (± 0.04)
Disconjugate Post-saccadic drift 160 ms (°)	−0.03 (± 0.13)	−0.05 (± 0.10)	−0.05 (± 0.13)	−0.03 (± 0.13)
Disconjugate Post-saccadic drift 160 ms (°) Absolute value	0.15 (± 0.08)	0.13 (± 0.05)	0.14 (± 0.07)	0.14 (± 0.06)
Conjugated Post-saccadic drift 80 ms (°)	−0.09 (±0.05)	−0.09 (±0.07)	−0.10 (±0.06)	−0.11 (±0.07)
Conjugated Post-saccadic drift 80 ms (°) Absolute value	0.14 (± 0.05)	0.13 (± 0.06)	0.14 (± 0.05)	0.14 (± 0.06)
Conjugated Post-saccadic drift 160 ms (°)	−0.13 (±0.06)	−0.11 (±0.08)	−0.13 (±0.08)	−0.14 (±0.08)
Conjugated Post-saccadic drift 160 ms (°) Absolute value	0.20 (± 0.07)	0.18 (± 0.07)	0.19 (± 0.07)	0.19 (± 0.07)
**(B)—REGRESSIVE READING SACCADES**				
Saccades amplitude (°)	−1.92 (± 0.45)	−1.94 (± 0.55)	−1.84 (± 0.38)	−1.69 (± 0.18)
Fixation duration (ms)	243.09 (± 53.31)	231.26 (± 57.03)	234.33 (± 65.51)	241.61 (± 62.93)
Disconjugacy of saccades (°)	0.07 (± 0.10)	0.11 (± 0.12)	0.08 (± 0.07)	0.10 (± 0.09)
Disconjugacy of saccades (°) Absolute value	0.16 (± 0.09)	0.17 (± 0.10)	0.13 (± 0.06)	0.14 (± 0.07)
Disconjugate Post-saccadic drift 80 ms (°)	−0.06 (± 0.08)	−0.03 (± 0.07)	−0.07 (± 0.04)	−0.07 (± 0.09)
Disconjugate Post-saccadic drift 80 ms (°) Absolute value	0.12 (± 0.05)	0.11 (± 0.03)	0.09 (± 0.02)	0.12 (± 0.07)
Disconjugate Post-saccadic drift 160 ms (°)	−0.09 (± 0.10)	−0.03 (± 0.13)	−0.08 (± 0.06)	−0.07 (± 0.07)
Disconjugate Post-saccadic drift 160 ms (°) Absolute value	0.16 (± 0.07)	0.14 (± 0.06)	0.12 (± 0.04)	0.13 (± 0.08)
Conjugated Post-saccadic drift 80 ms (°)	0.14 (±0.18)	0.06 (±0.31)	0.10 (±0.08)	0.11 (±0.11)
Conjugated Post-saccadic drift 80 ms (°) Absolute value	0.17 (± 0.18)	0.23 (± 0.23)	0.13 (± 0.08)	0.15 (± 0.09)
Conjugated Post-saccadic drift 160 ms (°)	0.17 (±0.17)	0.02 (±0.63)	0.10 (±0.10)	0.13 (±0.13)
Conjugated Post-saccadic drift 160 ms (°) Absolute value	0.22 (± 0.16)	0.37 (± 0.51)	0.18 (± 0.09)	0.18 (± 0.13)
**(C)—GLOBAL READING TIME**				
Global reading time (s)	83.11^*^^+^(±20.19)	73.44^+^(±18.66)	72.00^*^ (±19.97)	67.00 (±17.46)

* *Significant long term effect (between beginning of the 2 sessions or between end of the 2 sessions)*.

+ *Significant short term effect (between the first and the second reading trial of one session)*.

**Figure 8:**
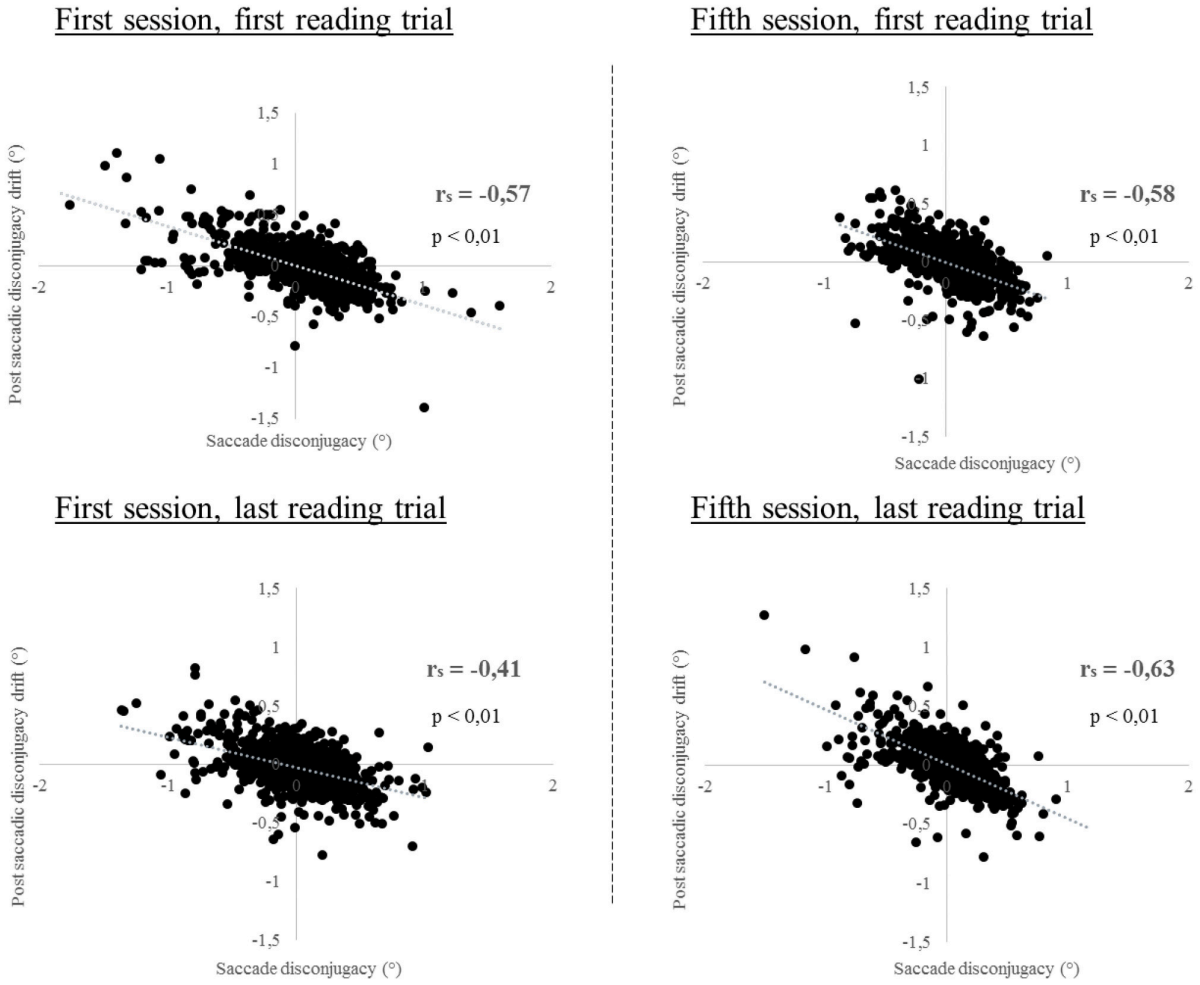
**Linear regression plot of the amplitude of saccade disconjugacy in degrees (°) as a function of the amplitude of the following post-saccadic disconjugacy drift in degrees (°) measured 80 ms after the end of each progressive reading saccade**. The entire sample has been plotted for each reading trial. Spearman r_*s*_ correlation coefficient and *p*-values are indicated.

For the majority of the subjects (between 67 and 89%, depending on the reading trial), the amplitude of the saccade disconjugacy was negatively and significantly (*p* < 0.05) correlated with the amplitude of the post-saccadic disonjugacy drift, measured at 80 or 160 ms after the end of the saccade; thus the post-saccadic drift may act to reduce the misalignment of the eyes at the end of the saccade (see Vernet and Kapoula, 2009). The Spearman r_s_ correlation coefficient values are shown on Table 4 for each subject and for each reading trial of the first and the fifth session, significant values (*p* < 0.05) are written in bold type. When studying the same parameters (80 ms) for all the subjects together, a significant correlation is observed for each reading trial (*p* < 0.001, see Figure 8) which confirms the individual data.

**Table 4:** **Spearman r_s_ correlation coefficient between the amplitude of the saccade disconjugacy and the post-saccadic disconjugacy drift calculated 80 and 160 ms after the end of the saccade**.

**Subject**	**First trial, first session**	**Second trial, first session**	**First trial, fifth session**	**Second trial, fifth session**
**SPEARMAN r_s_ CORRELATION COEFFICIENT VALUES (80 ms DRIFT)**				
s1	**−0.69**	**−0.57**	**−0.59**	**−0.75**
s2	**−0.49**	**−0.57**	**−0.24**	**−0.59**
s3	**−0.79**	**−0.61**	**−0.44**	**−0.42**
s4	0.1	−0.14	**−0.28**	**−0.17**
s5	**−0.33**	0.02	**−0.22**	−0.09
s6	**−0.23**	**−0.44**	**−0.37**	**−0.24**
s7	**−0.34**	**−0.4**	**−0.38**	**−0.55**
s8	**−0.52**	**−0.32**	**−0.62**	**−0.39**
s9	**−0.44**	**−0.56**	**−0.63**	**−0.68**
**SPEARMAN r_s_ CORRELATION COEFFICIENT VALUES (160 ms DRIFT)**				
s1	**−0.62**	**−0.57**	**−0.64**	**−0.72**
s2	**−0.28**	**−0.37**	−0.11	**−0.53**
s3	**−0.77**	**−0.61**	**−0.34**	**−0.37**
s4	0.07	−0.15	**−0.27**	−0.16
s5	**−0.29**	0.07	−0.17	−0.09
s6	**−0.25**	**−0.26**	**−0.33**	**−0.22**
s7	**−0.36**	**−0.39**	**−0.28**	**−0.56**
s8	**−0.39**	−0.17	**−0.47**	**−0.28**
s9	**−0.28**	**−0.55**	**−0.49**	**−0.64**

*Significant values (p < 0.05) are written in bold type*.

#### Short terms effects

##### Progressive reading saccades and fixations

No significant changes were found in terms of saccade amplitudes, fixation durations or conjugate and disconjugate post-saccadic drifts (*p* > 0.05).

##### Regressive reading saccades and fixations

There was a difference in terms of numbers of regressive saccades between the first and the second reading trial of each session (41 ± 18 vs. 37 ± 14 for the first session, 30 ± 14 vs. 27 ± 15 for the last session). Yet, this reduction was not statistically significant (respectively *Z* = 0.89, *p* = 0.37 and *Z* = 1.12, *p* = 0.26). No significant changes were found in terms of saccade amplitudes, fixation durations or conjugate and disconjugate post-saccadic drifts (*p* > 0.05).

#### Long terms effects

##### Progressive saccades

The non-parametric Friedman ANOVA test showed a significant effect of the tested conditions on the absolute value of the disconjugacy of progressive saccades [${\text{X}}_{\left(9,\;3\right)}^{2}$ = 10.47, *p* = 0.015]. There was a decrease in terms of mean value of the disconjugacy after the vergence rehabilitation. The non-parametric Wilcoxon test showed a significant decrease between the first reading trial of the two sessions (0.21 ± 0.10° vs. 0.15 ± 0.08°, *Z* = 2.67, *p* = 0.008). Additionally, after ranking the data, the two-way ANOVA test confirmed the results below: the main effect for long terms effect of vergence rehabilitation yielded an F ratio of *F*_(1, 8)_ = 14.23, *p* = 0.0054.

The non-parametric Friedman ANOVA test showed significant effect of the tested conditions on fixation duration [${\text{X}}_{\left(9,\;3\right)}^{2}$ = 10.20, *p* = 0.017]. The non-parametric Wilcoxon test showed a significant decrease of the fixation duration between the trials of the two sessions, and this is the case either for the first reading trial (286.46 ± 59.97 ms vs. 266.79 ± 56.70 ms, Z = 2.67, *p* = 0.008) and the second reading trial (269.92 ± 51.08 ms vs. 258.33 ± 55.38 ms, *Z* = 2.31, *p* = 0.021), comparing the first and the fifth session of rehabilitation. Additionally, after ranking the data, the two-way ANOVA test confirmed the results below: the main effect for long terms effect of vergence rehabilitation yielded an F ratio of *F*_(1, 8)_ = 30.76, *p* = 0.00054.

##### Regressive saccades

When comparing first and fifth rehabilitation sessions, we observed a decrease of the number of regressive saccades both for the first reading trial of the two sessions (41.11 ± 18.29 vs. 30.22 ± 14.18) and for the second reading trial of the two sessions (37 ± 14.74 vs. 27.11 ± 15.49). Comparisons between the first reading trial of the two sessions showed a significant difference (*Z* = 2.25, *p* = 0.024). After ranking the data, the two-way ANOVA test confirmed the results below with a significant the main effect concerning long terms effects: *F*_(1, 8)_ = 12.903, *p* = 0.0071. Yet, comparison between the last reading trial of the two sessions tended to give a similar result but was not statistically significant (*Z* = 1.95, *p* = 0.05).

No significant changes were found in terms of saccade amplitudes, fixation durations or conjugate and disconjugate post-saccadic drifts concerning long term effects of rehabilitation (*p* > 0.05).

#### Global reading time

Mean values and standard deviations for the group of nine subject and for each testing condition are shown on Table 3C. The non-parametric Friedman ANOVA test showed an effect of the condition on the global time of reading of the text *l'Alouette* [${\text{X}}_{\left(9,\;3\right)}^{2}$ = 14.97, *p* = 0.0019].

#### Short terms effects

Comparison between the first (83.11 ± 20.19 s) and the second reading trial (73.44 ± 18.66 s) of the first session showed a significant decrease in terms of time for the reading task *l'Alouette* (*Z* = 2.25, *p* = 0.024). After ranking the data, the two-way ANOVA test confirmed the results below: the main effect for short terms effect of vergence rehabilitation yielded an F ratio of *F*_(1, 8)_ = 12, *p* = 0.0085. Yet, no significant decrease was found for the two reading trials of the last session (72.0 s ± 19.97 vs. 67.0 s ± 17.46), run after the vergence rehabilitation (*Z* = 1.54, *p* = 0.12).

#### Long terms effects

Comparison between the first reading trial (83.11 ± 20.19 s) of the first session and the first reading trial of the fifth session (72 ± 19.97 s) showed a significant decrease of time for the reading task *l'Alouette* (*Z* = 2.67, *p* = 0.0076). After ranking the data, the two-way ANOVA test confirmed the results below: the main effect for long terms effect of vergence rehabilitation yielded an F ratio of *F*_(1, 8)_ = 21.33, *p* = 0.0017. Yet, no significant differences were found between the second reading trial of the first and the fifth session (73.44 ± 18.66 s vs. 67.0 ± 17.46 s; *Z* = 1.54, *p* = 0.12).

A multiple linear regression analysis was used to develop a model for predicting the global reading time value of the subjects from their individual absolute mean value of saccade disconjugacy and their fixation duration mean value. Basic descriptive statistics and regression coefficients are shown in Table 5. As expected, the fixation duration appears to be a better predictor of the global reading time value, as they are significantly and positively correlated in the first trial of the first session and in both trials of the fifth session (*p* < 0.02): the shorter the fixation, the faster the reading task was accomplished. This was not the case concerning saccade disconjugacy (*p* > 0.05, see Table 5).

**Table 5:** **Results of the Multiple Linear Regression Analysis (MLRA), as a model to predict the global time of reading from the mean values of the fixation duration with the absolute mean values of saccade disconjugacy**.

**Chronology**	**Variables**	**B**	**SE B**	**β**	**SE β**	**p**
First trial, first session	MLRA	*R*^2^ = 0.679, *F*_(2, 6)_ = 6.373, *p* = 0.033^*^				
	Duration	0.28	0.08	0.83	0.24	0.0129^*^
	Disconjugacy	3.81	47.12	0.02	0.24	0.94
Last trial, first session	MLRA	*R*^2^ = 0.457, *F*_(2, 6)_ = 2.530, *p* = 0.159				
	Dur.	0.25	0.11	0.69	0.31	0.0705
	Disconj.	7.41	58.92	0.04	0.31	0.90
First trial, fifth session	MLRA	*R*^2^ = 0.731, *F*_(2, 6)_ = 8.147, *p* = 0.019^*^				
	Dur.	0.25	0.07	0.85	0.22	0.0081^**^
	Disconj.	8.00	48.30	0.04	0.22	0.87
Last trial, fifth session	MLRA	*R*^2^ = 0.692, *F*_(2, 6)_ = 6.742, *p* = 0.029^*^				
	Dur.	0.27	0.07	0.85	0.23	0.0107^*^
	Disconj.	−30.12	61.38	−0.11	0.23	0.64

Significance is indicated with an asterisk for p < 0.05 (^*^) or twice for p < 0.01.

To summarize the main results, mean values of difference between conditions and associated standard deviation concerning the main reading parameters that significantly change are shown on Table 6.

**Table 6:** **Mean values and SD of the difference between the conditions concerning long term and short term effects**.

		**Short term effect (First session)**	**Short term effect (Last session)**	**Long term effect (First trials)**	**Long term effect (Last trials)**
Global Time of reading (s)	Mean SD	−9.67 (±9.54)	−5.00 (±7.55)	−11.11 (±9.94)	−6.44 (±11.23)
Fixation duration (ms)	Mean SD	−16.54 (±31.98)	−8.46 (±21.38)	−19.67 (±17.59)	−11.60 (±11.01)
Disconjugacy of saccades (°, absolute value)	Mean SD	−0.02 (±0.06)	0.01 (±0.03)	−0.06 (±0.06)	−0.03 (±0.07)
Numbers of regressive saccades	Mean SD	−4.11 (±13.21)	−3.11 (±11.58)	−10.89 (±11.68)	−9.89 (±12.94)

#### Control group

An additional reading test was run two times on three control students. The results shown a decrease of global reading time by 2 s when tested twice) after a 35 min interval (77 ± 5 s for the first trial vs. 75 ± 8 s for the second trial, using an interval similar to that used in the main study).

## Discussion

### Summary of results

The study shows a few short term effects and mostly long term effects of vergence rehabilitation. The vergence double-step protocol improved vergence accuracy and reduced the variability, tested in a regular single step vergence test. This behavior indicates a central oculomotor neuroplasticity, similarly to that described in numerous studies for saccade adaptation (see Alahyane and Pélisson, 2005) and for vergence (Takagi et al., 2001; Kapoula et al., 2016). Our present study shows lasting beneficial effects, i.e., in accuracy and concerning variability, between first and last session. Moreover values became similar or even better to those from control subjects and provide evidence for a real rehabilitation method.

More precisely, significant changes induced by a single session of vergence rehabilitation concern two parameters: (1) the global reading time, with a significant decrease between the two reading trials run in the first session of vergence rehabilitation; (2) the gain in divergence movement that increased for the second trial compared to the first run in the final session of vergence rehabilitation.

In addition, the complete vergence rehabilitation led to several significant findings: (1) the symptomatology decreased; (2) convergence at far distance measured by orthoptics tests increased; (3) the gain of convergence and divergence movements measured with oculography increased and the variability of the movements' amplitude decreased; (4) the global reading time decreased between the sessions; (5) the duration of the fixations during reading decreased; (6) the absolute value of the disconjugacy of reading saccades decreased; (7) the numbers of regressive reading saccades decreased.

### Short term effects: increase of reading speed

Short term effects of vergence rehabilitation were rather weak: the vergence tested before and after 35 min of vergence training only showed a significant increase of the gain in divergence movement in the fifth session (the gain increased by 0.06 ± 0.06). However, there was a tendency for reducing the variability of the convergence and the divergence response, particularly during the first session (respectively reduced by 8 ± 26% and 19 ± 32%). Additionally, there is a significant decrease of the global reading time, which reduced by about 10 s for the first session of vergence rehabilitation, and that was the case only for the first session. Similar decrease was visible in the last session, but to a lesser amount (5 s only) and the change did not reach statistical significance. It is therefore interesting to investigate whether how reading speed increased, as it could be attributable to a simple training effect. However, the additional reading test on control students showed a decrease of reading time by 2 s when tested twice) after a 35 min interval (77 ± 5 s for the first trial vs. 75 ± 8 s for the second trial), showing a potential limited training effect comparing with the decrease of the main study. Additionally, the parameters of the saccades for both progressive and regressive, or fixations duration, did not show statistical short term significance modulation in the first and the fifth session. Even though, the value of fixation durations and of saccades disconjugacy modulated more within the first session than within the last session, after the complete vergence rehabilitation. Overall, one could summarize that, weak short term modulation of the vergence properties corresponds to weak modulations of properties of saccades and fixations during the reading task. The significant decrease of the global reading time within the session is the only benefit and could be attributable to vergence exercising. Consequently, we argue that in parallel with a training effect, a small fraction of the reduction of reading time is due to improvement of saccade binocular coordination leading to shorter fixation duration: the subjects improved their reading speed, possibly due to a combination of reducing their intra-saccadic disconjugacy and their fixation duration (discussed below for long term effects). The fact that short term modulations were present only for the first session sheds light on the nature of vergence disorders. In other words, short term effects appear only when the vergence system is not yet completely rehabilitated. After the four sessions of rehabilitation, the vergence system was rather stabilized with no changes occurring further within the fifth session. The important modulations of vergence rehabilitation comparing the first and the fifth session were in terms of long term effect and occurred after the entire 5 session program of rehabilitation was completed, and are discussed below.

### Long term effects: decrease of the saccade disconjugacy

In the present study, after a 5 session vergence rehabilitation using a research based method, a number of positive evolutions has been shown: subjectively, symptoms significantly dropped down, associated with increase of the gain of convergence and divergence movements (respectively increased by 0.24 ± 0.25 and 0.18 ± 0.24 when comparing the beginning of each session, see Figure 5) but also their regularity (respectively decreased by 23 ± 25% and 22 ± 30%), and increased vergence capacities measured with a prism bar. The significant decrease of the CISS score after the vergence rehabilitation could represent at least partially effects of care taking and is not significantly correlated with the increase of the vergence gain (see Figure 5). However, the limited number of subjects call for further research with a larger sample concerning this correlation. The study shows the clinical validity of the REMOBI and the efficiency of the rehabilitation with only 5 sessions of 35 min each, i.e., a total amount of 2200 vergence movements. These results have been presented more extensively and discussed in another study (Kapoula et al., 2016). Here we focus on the effects of vergence rehabilitation on saccade disconjugacy, which is an indicator of the quality of the coordination of saccades. Optimal coordination of saccades implies that both eyes fixate properly the same letter which enables single binocular vision, facilitating subsequent processing of reading. At a theoretical level, it has been proposed that perfect binocular coordination of the saccades required during reading is achieved by learning and neuroplasticity (Bucci et al., 2008; Vernet et al., 2008).

It has been hypothesized that this neuroplasticity involves a capacity to program an appropriate small and fast vergence command that occurs rapidly within the saccade. This vergence command will help to reduce the primary saccade disconjugacy which would result from the asymmetry of innervational circuits and peripheral muscle of the abducting adducting circuits (Bucci and Kapoula, 2006). Given that the gain of the vergence along the medial plan increased in the present study, we could infer that the intra-saccadic vergence needed to make the saccade more equal was also improved. Our observations of reduced saccade disconjugacy after the vergence rehabilitation provide convincing evidence for such hypothetical mechanism. The present study confirms such neural plasticity in young adults, and in only 5 sessions of rehabilitation. Nevertheless, the limited number of subjects and their variability concerning saccade disconjugacy when reading call for further research involving a larger sample of subjects showing vergence disorders. Thus, a possible classification using the gains in convergence and divergence recorded with video-oculography could be helpful to understand the impact of such disorders on the coordination of reading saccades and to build an efficient rehabilitation program adapted to each subgroup. Next, we will discuss the effects on reading speed.

### Reduction of fixation duration

**Figure 9:**
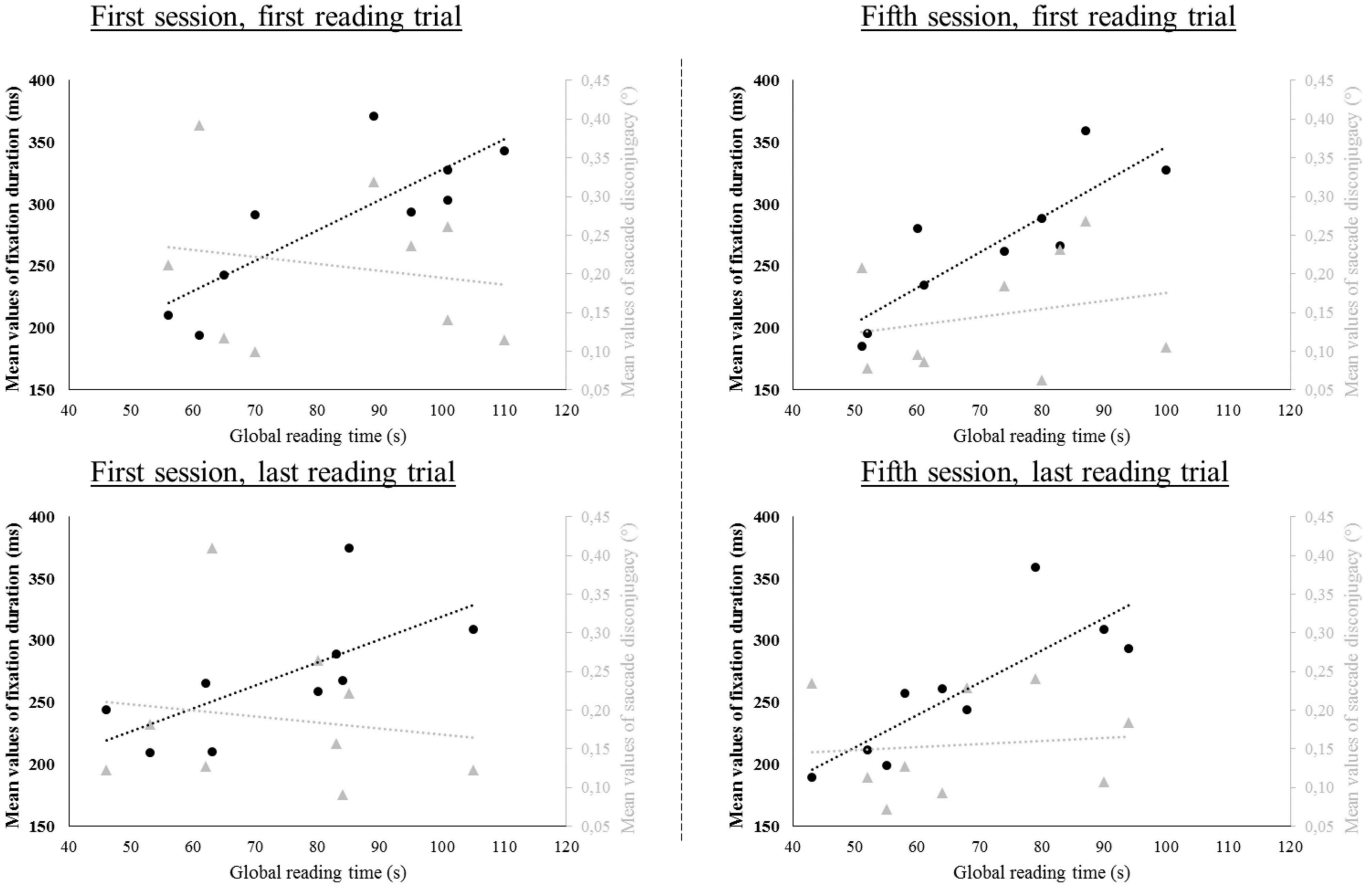
**Linear regression plot of the mean values of the amplitude of saccade disconjugacy in degrees (°, light gray marks) and of the mean values of the fixation duration in milliseconds (ms, black marks) as a function of the global time of reading in seconds (s)**. The individual mean values have been plotted for each reading trial and trend lines have been added for each variable.

The major result here is the decrease of the fixation durations. This effect results from several hypothetically parallel processes improved after vergence rehabilitation. Several studies showed that fixation duration in reading is influenced by linguistic factors like the frequency of the fixated word, the predictability of the fixated word or its semantic relation with the prior other words (Rayner, 1998; Rayner et al., 2006). However, the *Alouette* test was firstly designed to detect dyslexia in children, with several non-common words in sentences that do not have any meaning. Thus, semantic relation between words are not possible, and word predictability or memorization are not facilitated, unlike with common texts. This test is one of the most reliable tests to evaluate reading performance in France and is widely used for diagnosis and follow up of dyslexic children (Valdois et al., 2003; Lobier et al., 2012). The students participating in this study were not dyslexic, yet it is important that the test used for reading should reliably evaluate reading performance (Sprenger-Charolles et al., 2009). We believe that we found similar results to the study of Wilkins et al. (1996) that shows a tight correlation in fixation duration between the first and second reading trials in the first and the last session, or even across sessions. Yet, even if this indicates reliable measure of reading, fixation duration is found to be slightly modulated by vergence rehabilitation. Even if a small training effect can appear with repetition, we argue that shorter fixation durations are partially due to motor improvement, e.g., in binocular coordination of saccades. After de vergence rehabilitation, we can see on Figure 9 an interesting inversion of tendency suggesting that when the mean values of saccade disconjugacy were lower, the global reading time became shorter, and that was not the case prior to rehabilitation. Even if the regression analysis did not show its significant impact, one could argue that a part of the reduction of fixation duration comes from a better coordination of the reading saccades. However, further studies are needed to consolidate such interpretation. To process visual information readily, fixation should be relatively stable with the eyes aligned to the same letter. Identification of a fixated word is almost an automatic process (Rayner, 1998). The important thing is that it is certain that words were recognized automatically as each word was fixated by the eyes. At least, the only parameters that changed were the fixation duration and the amplitude of the saccade disconjugacy, confirming that the amplitude of the saccades and the number of progressive saccades remained similar. Given that there was no statistical difference between reading trials in post-saccadic drifts but only in the absolute value of saccade disconjugacy, vision could be clear and single from the beginning of the fixation, i.e., from the offset of the saccade. There is early evidence that visual processing (Vernet and Kapoula, 2009) starts from the beginning of the fixation (e.g., first 48 ms); subsequent high level processes involved for reading could also start and achieved earlier, thereby leading to shorter fixation duration. One could also argue that with the eyes better aligned on the word, visual analysis and attention process are better thereby, facilitating the reading process. Such a subtle effect of vergence rehabilitation on the duration of reading fixation is reported for the first time; it reinforces the hypothesis that vergence is a vector of attention, linked with cognitive executive functions (Daniel and Kapoula, 2016). This result and the reduction of regressive saccades corroborate the interpretation above: reading becomes easier thereby, there is less need of making regressive saccades. This is another strong argument for a connection between quality of vergence and reading processes. Finally, saccade disconjugacy and post-saccadic drift disconjugacy were highly correlated for all reading tests and vergence rehabilitation did not modify this relationship. Moreover, this correlation was reinforced after the rehabilitation, shown by higher correlation coefficients in the two reading trials of the fifth session (respectively r_s_ = −0.58 and r_s_ = −0.63, see Figure 8). Our observations here are in line with those of Vernet et al. (2008). Such strong correlations are indicative of a central vergence mechanism subtending both the intra saccadic and post-saccadic disconjugacy.

## Conclusion

In conclusion, vergence rehabilitation with a double-step the research based method improves not only vergence but also binocular control of saccades and fixations during reading. Improvement of binocular coordination would contribute to improvement of single binocular vision and thus to more focused attention which is necessary for reading.

## Author contributions

All authors listed, have made substantial, direct and intellectual contribution to the work, and approved it for publication.

### Conflict of interest statement

The authors declare that the research was conducted in the absence of any commercial or financial relationships that could be construed as a potential conflict of interest.
